# Plasma metabolomics profiles indicate sex differences of lipid metabolism in patients with Parkinson’s disease

**DOI:** 10.1038/s41598-024-82674-3

**Published:** 2024-12-28

**Authors:** Ling Hu, Yuan-Jun Huang, You-Dong Wei, Tao Li, Wei Ke, Guang-Hui Chen, Mei-Xue Dong

**Affiliations:** 1 Department of Neurology, Hubei General Hospital, Renmin Hospital of Wuhan University, Wuhan, Hubei China; 2 The First Branch, The First Affiliated Hospital of Chongqing Medical University, Chongqing, China; 3 Department of Neurology, The First Affiliated Hospital of Chongqing Medical University, Chongqing, China; 4 Department of Pharmacy, Hubei General Hospital, Renmin Hospital of Wuhan University, No.238 Jiefang Road, Wuchang District, Wuhan, 430060 Hubei China

**Keywords:** Metabolomics analysis, Sex difference, Parkinson’s disease, Fatty acids and conjugates, Eicosanoids, Glycerophosphocholines, Neuroscience, Medical research, Neurology

## Abstract

The effect of sexual dimorphism on the metabolism of patients with Parkinson’s disease has not been clarified. A group of patients with Parkinson’s disease and healthy controls were recruited, and their clinical characteristics and plasma were collected. Untargeted liquid chromatography-mass spectrometry-based plasma metabolomics profiling was performed. Differentially expressed metabolites between patients and healthy controls were respectively identified in the male and female participants and metabolite set enrichment analyses were further employed. A total of 75 patients with Parkinson’s disease (37 males and 38 females) and 31 healthy controls (16 males and 15 females) were enrolled while no significant differences can be discovered in clinical characteristics. The constructed male-specific metabolic model from orthogonal partial least squares-discriminant analysis can’t well recognize female patients and the female-specific model also can’t accurately identify male patients. There were 55 differentially expressed metabolites in the male participants, and fatty acids and conjugates and eicosanoids were the significantly enriched metabolite sets. Meanwhile, 86 metabolites were differentially expressed in the female participants while fatty acids and conjugates and glycerophosphocholines were enriched. Only 17 metabolites were simultaneously changed in both male and female patients. Significant sex differences of lipid metabolism were found in patients with Parkinson’s disease.

**Supplementary Information:**

The online version contains supplementary material available at 10.1038/s41598-024-82674-3.

## Introduction

Parkinson’s disease (PD) is the second most prevalent neurodegenerative disease in the world while its pathogenesis has not been clearly elucidated^1^. Currently, PD is recognized as an interplay of genetic and environmental factors, typically presenting as death of dopaminergic neurons in the substantia nigra pars compacta and decreased dopamine levels in striatum^2^. The clinical presentation of PD patients includes typical motor symptoms (resting tremor, bradykinesia, rigidity, and abnormal posture and gait) and inconspicuous non-motor symptoms (constipation, hyposmia, anxiety, depression, and rapid eye movement behavior disorder)^3^. The various symptoms of PD may contribute to the extensive alterations of neurotransmitters and brain regions while its mainstream treatment is still based on dopamine supplement^4^. Recent researches have focused on lipid metabolic disturbance and indicate lipid supplement (such as omega-3 polyunsaturated fatty acids) can be used for treating PD patients^5^.

Sexual dimorphism is a common natural phenomenon that can be found between different sexes in disease incidence, disease severity, metabolism, and pharmacodynamics of interventions. The prevalence rates of coronary heart disease, heart failure, stroke, and various metabolic syndromes are significant higher in the males while knee osteoarthritis mainly happens in the older females by a greater disease severity compared to the age-matched males^6^. Sexual dimorphism is reportedly due to sex chromosome and the following sex-specific hormone action. Increasing evidence points to obvious sex differences in the incidence and clinical characteristics of PD patients and risk of the male developing PD is twice as high as the female^7^. Altered levels of acetone and cholesterol in male PD patients and sex-related oxidative stress imbalance were also found in a metabolite and lipoprotein profiling study^8^. At homeostasis, the female is prone to incorporate free fatty acids into triglycerides whereas the male likely oxidizes circulating free fatty acids^9^. However, it is not clear whether sex differences in lipid and cholesterol metabolism can influence the occurrence and development of PD.

Exploring the pathogenesis of sex differences in PD patients is important for its treatment in the time of precision medicine. Metabolomics is a systematic qualitative and quantitative analysis of all metabolites in a biological sample, providing extensive biological information related with metabolism of organism for further analysis^10^. Metabolomics profiling of human plasma found that the sexual dimorphism of the metabolome may contribute to sex differences in stroke, hypertension, and chronic kidney disease^11^. Furthermore, in mouse models of Alzheimer’s disease metabolomics profiling reveals broad sex-specific metabolic differences in serum and brain metabolomes, just as human study cohorts^12^. Herein, we adopted untargeted liquid chromatography-mass spectrometry-based metabolomics profiling to analyze sex-specific metabolic changes and its possible mechanism in a Chinese population of PD patients.

## Results

### Clinical characteristics

After all, a total of 75 PD patients (37 males and 38 females) and 31 healthy controls (16 males and 15 females) were enrolled. The mean ages of the male and female participants between healthy controls and PD patients were without statistical differences, indicating each two groups of participants were comparable. It seemed that the prevalence of hypercholesterolemia (13.5% versus 43.8%) and mean level of Apo-B (0.83 ± 0.04 versus 0.99 ± 0.07) were lower in the male patients compared to the male controls while no statistical significances were found. There were also no significant differences in the other medical histories and lipid levels between each two groups (Table 1).

**Table 1:** Clinical characteristics of PD patients and healthy controls with different sexes included in this study.

Variable (SD/%)	HC-Male (16)	PD-Male (37)	*P* value	HC-Female (15)	PD-Female (38)	*P* value
Age (year)	62.56 (9.71)	66.84 (6.05)	0.253	65.73 (7.57)	65.45 (6.60)	1
Smoking (%)	9 (56.3%)	14 (37.8%)	0.331	0	0	-
Alcohol consumption (%)	4 (25%)	4 (10.8%)	0.315	0	0	-
Hypertension (%)	8 (50%)	13 (35.1%)	0.405	4 (26.7%)	11 (34%)	1
Diabetes mellitus (%)	1 (6.3%)	8 (21.6%)	0.315	1 (6.7%)	5 (14.2%)	0.963
Hypercholesterolemia (%)	7 (43.8%)	5 (13.5%)	0.119	2 (13.3%)	10 (26.3%)	0.885
CHD (%)	1 (6.3%)	2 (5.4%)	1	2 (13.3%)	8 (21.1%)	0.963
UPDRS	-	51.84 (4.26)	-	-	53.99 (3.99)	-
Hoehn-Yahr score	-	2.68 (0.20)	-	-	2.79 (0.19)	-
HAMD	-	11.51 (1.23)	-	-	13.42 (1.12)	-
HAMA	-	12.89 (1.20)	-	-	15.78 (1.15)	-
MMSE	-	25.51 (0.77)	-	-	23.95 (0.95)	-
TC (mmol/L)	4.52 (0.76)	4.16 (0.72)	0.253	4.33 (0.43)	4.55 (0.91)	0.859
TG (mmol/L)	1.74 (0.43)	1.26 (0.79)	0.119	1.41 (0.52)	1.24 (0.67)	0.859
HDL-c (mmol/L)	1.20 (0.34)	1.33 (0.36)	0.354	1.32 (0.32)	1.54 (0.38)	0.859
LDL-c (mmol/L)	2.98 (0.79)	2.59 (0.75)	0.253	2.83 (0.44)	2.79 (0.75)	1
Apo-A1 (g/L)	1.30 (0.21)	1.30 (0.22)	1	1.37 (0.24)	1.43 (0.23)	0.859
Apo-B (g/L)	0.99 (0.27)	0.82 (0.20)	0.119	0.87 (0.13)	0.87 (0.21)	1
Lpa (mg/L)	181.33 (243.89)	194.83 (255.79)	0.978	186.57 (281.95)	345.60 (384.23)	0.859
Weight (kg)	70.3 ± 2.7	62.5 ± 1.5	0.119	56.9 ± 2.3	55.0 ± 1.7	0.887
Height (cm)	166 ± 0.9	165.3 ± 1.1	0.843	154.1 ± 1.35	155.7 ± 0.8	0.859
BMI	25.5 ± 1.0	22.9 ± 0.6	0.119	24.0 ± 1.0	22.7 ± 0.7	0.859

PD, Parkinson’s disease; SD, standard deviation; HC, healthy control; CHD, coronary heart disease; UPDRS, Unified Parkinson’s disease rating scale; HAMD, Hamilton depression rating scale; HAMA, Hamilton anxiety rating scale; MMSE, mini-mental state examination; TC, total cholesterol; TG, triglyceride; HDL-c, high-density lipoprotein cholesterol; LDL-c, low-density lipoprotein cholesterol; Apo-A1, apolipoprotein A1; Apo-B, apolipoprotein; Lpa, lipoprotein a; BMI, body mass index; P values have already been adjusted using the Benjamini and Hochberg method.

## Metabolomics analysis

After excluding internal standards, a total of 10,403 individual peaks, including 6040 positive and 4363 negative peaks, were detected in approximately 98.8% of samples in each group. Based on the above peaks, orthogonal partial least squares-discriminant analysis (OPLS-DA) of all the participants were performed, indicating significant differences among the four subgroups, especially between the male and female PD patients (Supplementary Fig. 1).

**Fig. 1:**
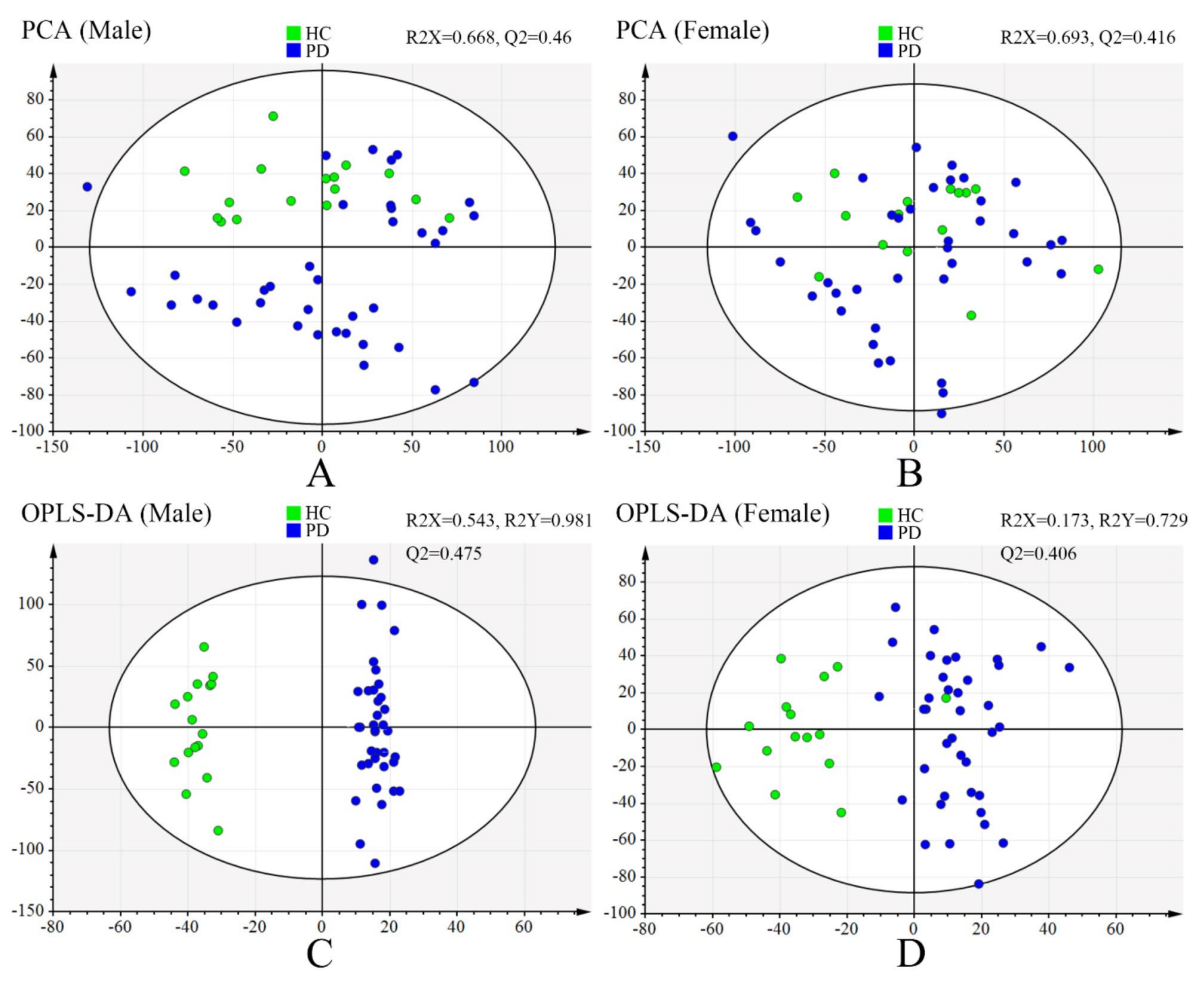
Multivariate statistical analyses of liquid chromatography-mass spectrometry-based metabolomics profiling between HCs and PD patients based on different sexes. (**A**) PCA score plot derived from metabolomics profiling of the male participants between HCs and PD patients. (**B**) PCA score plot of the female participants between HCs and PD patients. (**C**) OPLS-DA score plot of the male participants indicated clear separation between HCs and PD patients (R^2^X = 0.543, R^2^Y = 0.981, Q^2^ = 0.475). (**D**) OPLS-DA score plot of the female participants (R^2^X = 0.173, R^2^Y = 0.729, Q^2^ = 0.406). The outlier of HCs is a 61-year-old female without any history of smoking, drinking, CHD, hypertension, diabetes, and hyperlipemia. We suppose that the participant might be a potential PD patient in future. HC, healthy control; PD, Parkinson’s disease; PCA, principal component analysis; OPLS-DA, orthogonal partial least squares-discriminant analysis.

**Fig. 2:**
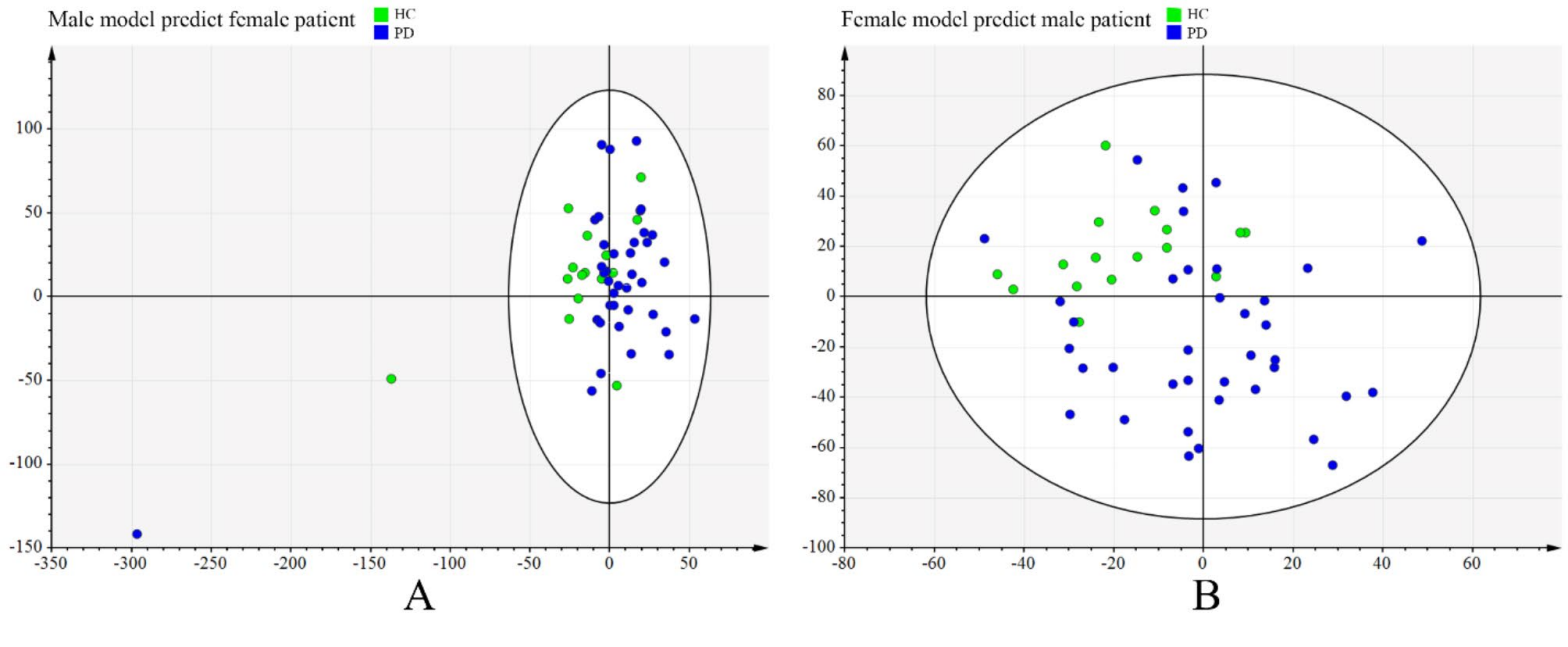
The predicted score plots based on constructed models for participants with different sexes. (**A**) The OPLS-DA model generating with male metabolites was used to predict class membership of female participants, showing 40% HCs was wrongly predicted as PD patients. (**B**) The OPLS-DA model generating with female metabolites was used to predict class membership of male participants, showing 37.5% HCs was wrongly predicted as PD patients. OPLS-DA, orthogonal partial least squares-discriminant analysis; HC, healthy control; PD, Parkinson’s disease.

**Fig. 3:**
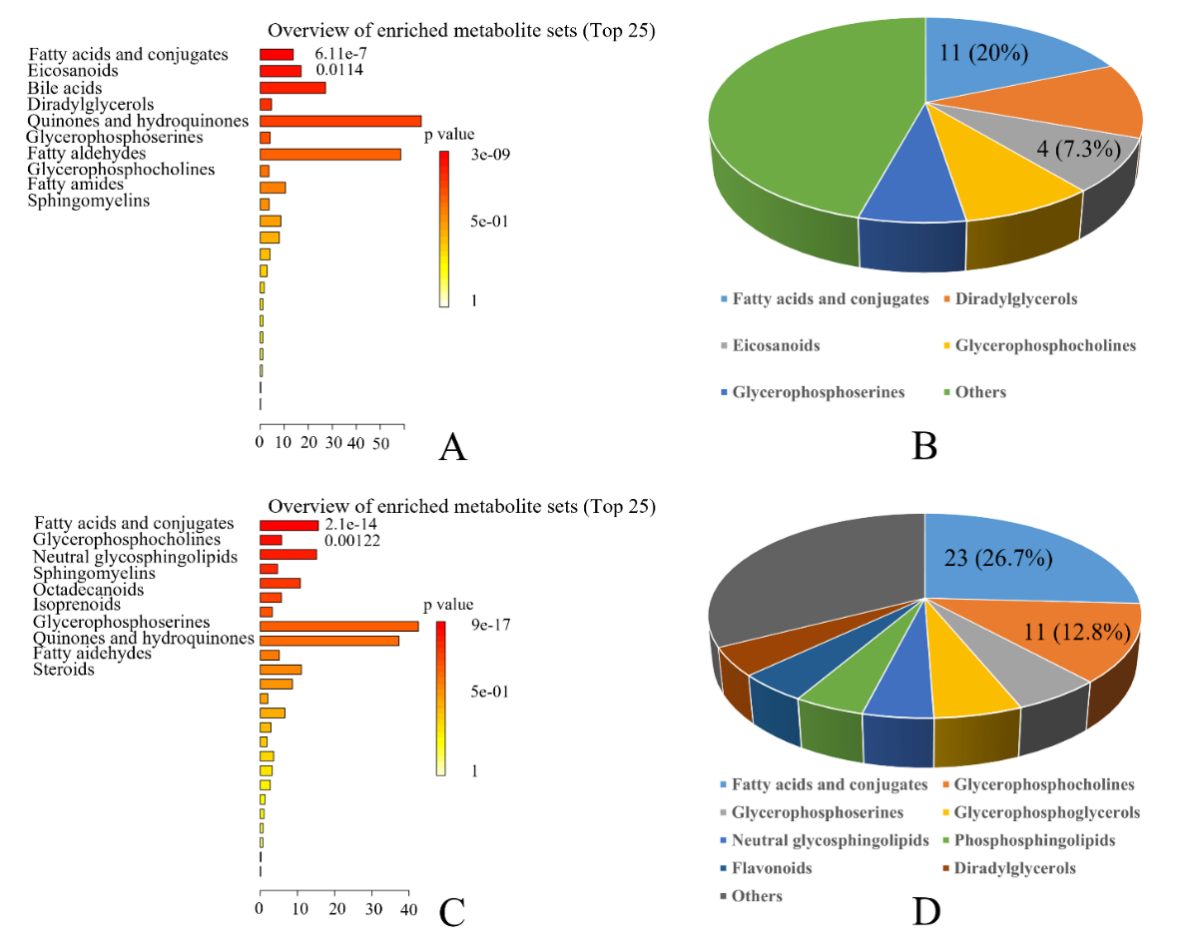
The overviews of enriched metabolite sets and categories of the differentially expressed metabolites in the male (Table 2) and female (Table 3) participants. (**A**) The metabolite set enrichment analysis indicated fatty acids and conjugates and eicosanoids were significantly enriched in the male participants. (**B**) There were 10 metabolites belonging to fatty acids and conjugates and 4 metabolites belonging to eicosanoids in the male participants. (**C**) The metabolite set enrichment analysis indicated fatty acids and conjugates and glycerophosphocholines were significantly enriched in the female participants. (**D**) There were 23 metabolites belonging to fatty acids and conjugates and 11 metabolites belonging to glycerophosphocholines in the female participants.

Score plots from principal component analysis (PCA) and OPLS-DA of the males were also performed. Eight components were found in the PCA, with a R^2^X value of 0.668 and Q^2^ value of 0.46 (Fig. 1A). Furthermore, the result of OPLS-DA showed clear separations between healthy controls and PD patients (R^2^X = 0.543, R^2^Y = 0.981, Q^2^ = 0.475) (Fig. 1C) while response permutation test indicated the model was stable and reliable (Supplementary Fig. 2A). To assess sex-specificity of the model, the above OPLS-DA model was used to predict class membership of the female participants. The T-predicted scatter plot demonstrated that female PD patients could not be effectively separated from female healthy controls, indicating 40% of healthy controls were wrongly predicted as PD patients (Fig. 2A). There were 55 differentially expressed metabolites between the two groups (Table 2). Fatty acids and conjugates (11, 20%) and eicosanoids (4, 7.3%) were significantly enriched in the following metabolite set enrichment analysis (Fig. 3A,B). The differentially expressed eicosanoids in the male participants were PGF2alpha methyl ether, 20-carboxy-LTB4, Prostaglandin D3, and 11-dehydro-2,3-dinor-TXB2.

**Table 2:** The male-specific differentially expressed metabolites between healthy controls and PD patients in untargeted liquid-chromatography mass-spectrometry analysis.

Compound ID	Common name	m/z	RT (min)	Ion mode	VIP	FC	*P* value	MS	FS	ME (ppm)
*LMFA08020138*	N-linoleoyl taurine	405.278	2.17	Positive	1.524	2.0395	0.007	38.5	0	0.033
HMDB00207	Oleic acid	327.255	6.789	Negative	1.003	4.051	0.014	37.5	0	1.385
LMFA01070021	Alchornoic acid	369.266	5.413	Negative	1.424	4.923	<0.001	35.8	0	2.704
LMFA01020012	15-methyl palmitic acid	288.289	3.725	Positive	1.755	0.194	0.038	39.3	0	-1.569
LMFA01170036	Heneicosanedioic acid	379.283	6.993	Positive	1.373	0.604	0.012	37.6	0	2.058
HMDB01043	Arachidonic acid	631.471	9.343	Positive	1.745	0.653	0.005	37	0	2.615
LMFA01020334	Mycolipanolic acid (C27)	425.401	8.159	Negative	1.56	0.49	0.024	36.7	0	2.132
*HMDB01999*	Eicosapentaenoic acid	301.218	8.555	Negative	1.406	0.382	0.041	37.9	0	0.900
*LMFA01170010*	2-methyl-dodecanedioic acid	487.329	5.131	Negative	1.693	0.553	0.013	39.2	0	2.580
LMFA01020107	2-methyl-2-dodecenoic acid	257.176	3.829	Negative	1.461	3.65	<0.001	37.2	0	-0.882
HMDB13123	4,7,10,13,16-Docosapentaenoic acid	329.249	8.749	Negative	1.266	0.622	0.037	39.3	0	0.904
*LMFA03010073*	PGF2alpha methyl ether	377.266	6.493	Positive	1.169	0.616	0.03	38.1	0	-0.861
LMFA03020016	20-carboxy-LTB4	411.204	4.432	Negative	1.084	1.721	0.011	36.7	0	2.758
HMDB03034	Prostaglandin D3	351.217	8.939	Positive	2.226	0.581	<0.001	38.2	0	0.201
LMFA03030013	11-dehydro-2,3-dinor-TXB2	341.197	6.801	Positive	2.227	0.65	<0.001	39.3	4.33	2.253
*HMDB10398*	LysoPC(22:0/0:0)	580.434	8	Positive	1.436	0.661	0.025	36.6	0	0.481
HMDB10382	LysoPC(16:0/0:0)	496.34	8.857	Positive	2.205	0.64	0.001	46.3	41.9	0.092
LMGP01070003	LPC(P-15:0)	975.642	6.227	Negative	2.031	0.634	<0.001	39	11.4	0.256
HMDB07952	PC(15:0/22:1)	822.599	9.284	Negative	2.337	0.656	<0.001	40.4	14.6	-0.010
*LMGP04010477*	PG(19:0/22:2)	889.617	9.983	Negative	1.443	0.522	0.025	36.1	0	-0.378
HMDB07951	PC(15:0/20:5)	764.524	8.548	Negative	1.328	0.664	0.034	38.2	3.5	0.753
*LMGP03010047*	PS(12:0/15:0)	666.434	6.608	Positive	1.103	1.609	0.01	37.2	0	0.248
*LMFA06000077*	2-tridecene-4,7-diynal	377.247	2.163	Positive	1.358	2.275	0.017	36.8	0	-2.326
*HMDB00020*	p-Hydroxyphenylacetic acid	153.055	1.622	Positive	1.906	199.695	<0.001	39.5	0	0.065
*HMDB11635*	p-Cresol sulfate	187.007	2.177	Negative	1.372	1.869	0.015	58.4	96	-1.997
*HMDB01858*	p-Cresol	107.05	2.177	Negative	1.195	1.891	0.041	39	0	0.705
*HMDB04667*	13 S-hydroxyoctadecadienoic acid	295.228	5.103	Negative	1.959	1.8126	<0.001	37.1	0	-1.321
*HMDB00748*	L-3-Phenyllactic acid	211.061	1.815	Negative	1.991	290.731	<0.001	38.7	0	-2.451
*LMPK12090001*	Tephcalostan	361.071	2.526	Negative	1.838	47.512	<0.001	37.6	0	-0.943
*LMGP10030015*	PA(P-16:0/18:4)	653.454	8.802	Positive	1.373	0.516	0.049	36.7	0.429	-0.387
*LMSP0504AH02*	Aleb(d18:1/18:0)	875.998	6.945	Positive	1.984	0.291	0.005	32.2	0.055	2.939
LMGP03010028	PS(14:0/14:0)	680.45	7.067	Positive	1.741	1.594	<0.001	38.4	0	-0.102
HMDB00845	Neopterin	271.115	2.163	Positive	1.468	1.57	0.001	38.5	0	1.633
LMSP02010024	Cer(d18:2/16:0)	536.503	6.746	Positive	1.193	1.503	0.007	36.7	0	-1.964
HMDB00637	Chenodeoxycholic acid glycine conjugate	448.307	4.295	Negative	1.079	4.076	0.006	56.8	85.4	0.576
*HMDB00518*	Chenodeoxycholic acid	391.286	4.157	Negative	1.231	1.892	0.005	39.3	0	0.576
LMFA05000016	13-tetradecen-2,4-diyn-1-ol	249.149	5.796	Negative	1.722	1.568	<0.001	39.1	0.408	-1.675
LMPR02010037	plastoquinol-1	389.271	4.63	Negative	1.13	5.262	0.005	35.9	0	2.124
LMST02010040	Estradiol dipropionate	791.45	6.225	Positive	2.142	0.474	0.001	35.1	0	0.516
LMGL02040001	DG(P-14:0/18:1)	573.486	8.967	Positive	1.733	0.564	0.005	37.7	17.6	1.515
HMDB07065	DG(14:1/24:1/0:0)	671.559	10.02	Positive	1.518	0.533	0.015	36.4	0	0.956
HMDB07017	DG(14:0/18:3/0:0)	563.467	9.26	Positive	1.618	0.667	0.032	41.6	27.6	-0.673
LMGP03010022	PS(10:0/10:0)	284.666	6.06	Positive	1.666	0.583	0.009	37.6	0	1.899
HMDB07025	DG(14:0/20:4/0:0)	611.464	8.719	Positive	1.366	0.546	0.044	36.8	0	-1.325
LMSP02050005	CerP(d18:1/20:0)	696.53	9.412	Positive	1.971	0.389	0.003	36.6	0	-1.122
HMDB07029	DG(14:0/22:1/0:0)	645.541	10.061	Positive	1.604	0.65	0.008	37.6	0	-2.657
HMDB07014	DG(14:0/18:1)	589.479	9.3156	Positive	1.466	0.653	0.022	37.8	0	-2.930
HMDB07022	DG(14:0/20:2/0:0)	610.54	9.35	Positive	1.367	0.645	0.022	33.4	2.57	-1.220
LMSP03010022	SM(d18:0/22:0)	833.676	10.194	Negative	1.476	0.404	0.015	37.5	4.58	0.739
LMGP06050023	PI(22:2/0:0)	697.358	8.138	Negative	1.614	0.647	0.01	36	0	0.769
LMGL02010361	DG(31:2)	549.454	8.296	Negative	1.232	0.609	0.049	37.3	0	2.355
LMSP03010046	SM(d18:0/17:0)	717.593	9.407	Negative	2.326	0.354	0.001	37.2	0	1.242
HMDB00472	5-Hydroxy-L-tryptophan	485.169	2.26	Negative	2.292	0.607	<0.001	39.2	0	1.663
HMDB09195	PE(18:4/18:4)	776.452	7.851	Negative	1.26	0.598	0.022	35.5	0.964	1.864
LMGP03050014	PS(22:4/0:0)	594.282	5.94	Negative	1.513	0.549	0.017	38.2	0	1.251

Compound ID was mainly based on the Human Metabolome Database (www.hmdb.ca) and LIPID MAPS (www.lipidmaps.org); FC value was calculated as the ratio of the average mass response (area) between the two groups (FC value = PD/HC). P values less than 0.05 indicated significantly differences between the two groups. RT, retention time; VIP, variable influence on projection; FC, fold change; MS, matching score; FS, fragmentation score; ME, mass error; PD, Parkinson’s disease; HC, healthy control; P values have already been adjusted using the Benjamini and Hochberg method. Compound IDs underlined were shared differentially expressed metabolites in both sexes.

**Table 3:** The female-specific differentially expressed metabolites between healthy controls and PD patients in untargeted liquid-chromatography mass-spectrometry analysis.

Compound ID	Common name	m/z	RT (min)	Ion mode	VIP	FC	*P* value	MS	FS	ME (ppm)
*LMFA08020138*	N-linoleoyl taurine	405.278	2.17	Positive	1.599	1.902	0.01	38.5	0	0.033
*LMFA01170010*	2-methyl-dodecanedioic acid	487.329	5.131	Negative	2.688	0.371	0.008	39.2	0	2.580
LMFA01040054	methyl 8-[3,5-epidioxy-2-(3-hydroperoxy-1-pentenyl)-cyclopentyl]-octanoate	419.24	3.876	Positive	1.807	2.7	0.002	38.7	0	-0.764
LMFA01170057	9-hydroxy-hexadecan-1,16-dioic acid	303.217	8.588	Positive	1.713	0.587	0.017	39.2	0	-0.199
LMFA01150004	3-carboxy-4-methyl-5-propyl-2-furanpropanoic acid	503.19	7.87	Positive	1.7	1.782	0.004	37.9	0	2.198
LMFA01040038	methyl-10-hydroperoxy-8E,12Z,15Z-octadecatrienoate	342.264	3.438	Positive	1.382	1.989	0.015	38.7	0	-0.924
LMFA01030186	4,8,12,15,19,21-tetracosahexaenoic acid	357.278	4.972	Positive	1.306	1.826	0.041	38.9	0	-1.692
LMFA07010122	Linolenyl palmitate	520.508	9.095	Positive	1.992	0.623	0.017	37.6	0	-0.782
LMFA00000003	N-(3-(hexadecanoyloxy)-heptadecanoyl)-L-ornithine	656.593	10.027	Positive	2.29	0.642	0.006	38.2	0	-0.598
HMDB02231	Eicosenoic acid	309.28	7.865	Negative	1.657	1.771	0.005	38.7	0	1.319
HMDB02226	Adrenic acid	331.264	7.241	Negative	1.725	1.583	0.006	39.2	0	0.613
HMDB06528	Clupanodonic acid	329.249	7.034	Negative	1.677	1.514	0.005	39.3	0	0.904
HMDB03229	Palmitoleic acid	253.217	6.7	Negative	1.765	1.771	0.005	39.1	0	-1.339
LMFA01050421	8Z-decen-4,6-diynoic acid	297.244	5.33	Negative	1.645	1.601	0.007	36.2	0	-0.248
LMFA01020203	7-methyl-6E-hexadecenoic acid	267.233	7.041	Negative	1.565	1.622	0.016	37.6	0	0.220
HMDB02925	8,11,14-Eicosatrienoic acid	305.249	7.206	Negative	1.666	1.799	0.008	39	0	0.172
HMDB02068	Erucic acid	337.312	8.303	Negative	1.749	2.262	0.001	37.7	0	2.295
HMDB04704	9,10-DHOME	313.239	5.583	Negative	1.387	1.52	0.013	37.3	0	1.296
LMFA01020102	2-methyl-2E-heptenoic acid	283.191	4.343	Negative	1.739	2.167	0.002	38.7	0	-0.061
LMFA01170038	Tricosanedioic acid	405.3	8.303	Negative	1.679	3.015	0.001	44.4	37.8	2.988
*HMDB01999*	Eicosapentaenoic acid	301.218	6.596	Negative	1.729	3.574	<0.001	37.9	0	0.900
LMFA01060186	7-oxo-11E-Tetradecenoic acid	479.338	4.898	Negative	1.732	0.617	0.035	37	0	0.859
LMFA01020001	17-methyl-6Z-octadecenoic acid	295.264	7.617	Negative	1.466	2.07	0.03	36.2	0	0.384
*LMFA03010073*	PGF2alpha methyl ether	377.266	6.493	Positive	1.795	0.57	0.05	39.1	0	-0.516
*HMDB10398*	LysoPC(22:0/0:0)	580.434	8	Positive	2.015	0.627	0.03	36.6	0	0.481
HMDB07930	PC(14:1/P-18:1)	714.544	9.254	Positive	1.902	0.551	0.048	36	0	0.900
LMGP01030103	PC(P-20:0/22:4)	872.648	9.85	Positive	1.509	2.334	0.018	34.3	0	-2.497
LMGP01010380	PC(10:0/10:0)	583.41	7.287	Positive	1.817	0.609	0.028	33.7	0	2.925
LMGP01020004	PC(O-1:0/16:0)	510.356	6.5	Positive	2.081	0.647	0.039	50.8	62.7	0.954
LMGP01020033	PC(O-16:0/18:0)	792.613	9.708	Negative	1.877	0.64	0.032	37.3	0	0.556
HMDB00564	PC(32:0)	778.561	8.624	Negative	2.082	0.646	0.016	37.9	0	0.490
LMGP01020068	PC(O-16:0/3:0)	582.379	7.131	Negative	2.024	0.605	0.026	38.7	0	1.665
HMDB07886	PC(36:0)	834.623	9.832	Negative	2.73	0.413	0.002	36.7	0	0.310
HMDB07949	PC(15:0/20:4)	812.544	9.563	Negative	2.33	0.666	0.009	38.1	0	-1.302
LMGP01050001	PC(13:0/0:0)	452.279	5.151	Negative	1.652	0.619	0.021	39.6	0	0.823
*LMPK12090001*	Tephcalostan	361.071	2.526	Negative	2.038	36.46	<0.001	37.6	0	-0.943
*LMGP03010047*	PS(12:0/15:0)	666.434	6.608	Positive	2.608	1.949	<0.001	37.2	0	0.248
*LMFA06000077*	2-tridecene-4,7-diynal	377.247	2.163	Positive	1.561	2.34	0.01	36.8	0	-2.326
*HMDB00020*	p-Hydroxyphenylacetic acid	153.055	1.622	Positive	3.109	153.4	<0.001	39.5	0	0.065
*HMDB11635*	p-Cresol sulfate	187.007	2.177	Negative	1.631	1.867	0.009	58.4	96	-1.997
*HMDB01858*	p-Cresol	107.05	2.177	Negative	1.538	2.09	0.01	39	0	0.705
*HMDB04667*	13 S-hydroxyoctadecadienoic acid	295.228	5.103	Negative	2.553	1.901	<0.001	37.1	0	-1.321
*HMDB00748*	L-3-Phenyllactic acid	211.061	1.815	Negative	2.828	596.3	<0.001	38.7	0	-2.451
*LMGP04010477*	PG(19:0/22:2)	889.617	9.983	Negative	2.29	0.468	0.01	36.1	0	-0.378
*LMGP10030015*	PA(P-16:0/18:4)	653.454	8.802	Positive	2.162	0.44	0.016	36.7	0.429	-0.387
*LMSP0504AH02*	Aleb(d18:1/18:0)	875.998	6.945	Positive	1.995	0.419	0.009	32.2	0.055	2.939
HMDB06117	APGPR Enterostatin	519.264	8.301	Positive	1.516	3.099	0.004	37.3	0	-1.395
LMPK12120007	Isocordoin	309.148	6.698	Positive	1.622	2.096	0.019	38.7	0	-1.908
HMDB09776	PE(24:1/P-18:1)	829.681	9.767	Positive	3.217	0.277	0.002	34.6	0	2.639
HMDB11773	Cer(d18:1/14:0)	532.47	8.733	Positive	1.791	0.585	0.046	36.9	0	0.039
LMPK12060003	Amorphigenin O-vicianoside	722.264	9.987	Positive	2.253	0.646	0.007	32.7	0	-2.402
LMGP03010619	PS(20:3/22:6)	858.528	9.561	Positive	2.458	0.669	0.006	32.5	0	0.554
HMDB00253	Pregnenolone	655.469	9.034	Positive	2.091	0.565	0.016	36.6	0	-1.671
LMGP03010478	PS(19:0/22:4)	871.619	9.987	Positive	2.443	0.589	0.005	38.6	0.188	2.538
HMDB07032	DG(14:0/22:5/0:0)	637.48	8.802	Positive	1.932	0.603	0.024	39.3	1.13	0.228
LMPR02030027	2-demethylmenaquinone-8	720.572	8.767	Positive	2.239	0.616	0.016	32.9	0.09	0.485
*HMDB00518*	Chenodeoxycholic acid	415.282	4.972	Positive	1.378	1.953	0.032	35.1	0	-0.489
HMDB02014	cis-5-Tetradecenoylcarnitine	370.295	4.094	Positive	1.263	1.72	0.033	39	0	-1.001
HMDB01449	Allopregnanolone	319.263	7.335	Positive	1.955	1.787	<0.001	38.8	0	-0.039
LMGP03010527	PS(20:0/22:4)	885.633	10.199	Positive	2.001	0.463	0.015	36.3	0	-0.154
HMDB07031	DG(14:0/22:4)	639.494	9.04	Positive	2.016	0.62	0.021	44.7	25.3	-0.806
LMSP03010076	SM(d16:1/25:0)	823.663	10.199	Positive	2.019	0.659	0.013	52.2	66.3	-2.763
LMSP0501AB03	LacCer(d18:1/16:0)	884.601	8.767	Positive	2.584	0.554	0.007	37.5	1.44	-2.519
LMPR0106150012	3-oxoglycyrrhetinic acid	959.639	10.199	Positive	2.089	0.632	0.011	38.5	14.2	1.942
LMGL03012862	TG(14:1/20:5/20:5)	891.65	10.199	Positive	2.045	0.653	0.013	37.9	0	2.843
HMDB11775	Cer(d18:1/22:1)	642.578	9.822	Positive	2.148	0.669	0.009	38.3	0	-1.827
LMSP03010065	SM(d16:1/23:0)	795.632	9.774	Positive	2.566	0.594	0.006	48.7	48.4	-1.233
LMGP06010289	PI(18:0/22:2)	917.615	9.983	Negative	2.068	0.659	0.014	37.8	0	2.617
HMDB00641	L-Glutamine	145.062	1.904	Negative	1.976	2.07	<0.001	38.9	0	-0.155
HMDB01169	4-Aminophenol	263.103	1.904	Negative	2.064	2.028	<0.001	38.6	0	-1.062
LMSP03010006	SM(d18:1/22:0)	785.655	10.201	Negative	2.213	0.588	0.008	37.4	0.427	1.076
LMGP00000062	PGS	885.624	9.777	Negative	2.793	0.636	0.003	37.6	0	-1.819
LMSP03010005	SM(d18:1/20:0)	757.623	9.777	Negative	2.748	0.508	0.005	37.7	0	0.676
LMGP06010445	PI(19:0/22:2)	953.613	9.777	Negative	2.815	0.576	0.003	36.6	0	2.928
LMPR0104010007	Phytyl phosphate	751.542	9.825	Negative	1.949	0.411	0.019	38	0	0.990
LMGL03011824	TG(17:2/20:5/22:5)	957.711	7.351	Negative	1.461	1.797	0.04	36.2	0	1.947
LMGP04010699	PG(21:0/22:1)	873.657	9.77	Negative	2.356	0.347	0.008	33.9	0.894	-2.389
LMST01031092	Stoloniferone F	969.667	7.351	Negative	1.432	1.768	0.041	31.3	1.04	-0.650
LMGP06010214	PI(17:0/22:2)	903.596	9.777	Negative	2.667	0.353	0.005	35.4	0	-0.870
LMGP03010477	PS(19:0/22:2)	902.61	9.832	Negative	2.471	0.242	0.005	30.9	0	-2.902
LMST01010336	11-acetoxy-3beta,6alpha-dihydroxy-9,11-seco-5alpha-cholest-7-en-9-one	951.694	7.351	Negative	1.569	2.011	0.025	37.1	0	0.931
LMSP0501AA38	GlcCer(d18:2/23:0)	840.656	9.708	Negative	1.924	0.441	0.039	34.9	0	-0.911
LMSP0501AB05	LacCer(d18:1/20:0)	938.654	9.77	Negative	2.095	0.331	0.033	35.9	0	-1.296
LMPK12110281	Vitexin 3’’’,4’’’-Di-O-acetyl 2’’-O-rhamnoside	661.176	7.872	Negative	1.813	3.228	0.001	35.6	0	-2.089
HMDB07469	DG(20:3/22:6/0:0)	711.496	7.351	Negative	1.394	2.181	0.048	33.4	0.399	-1.644
LMGP20010005	PC(16:0/5:0(CHO))	638.368	6.303	Negative	1.772	1.975	0.003	36.5	0	0.441

Compound ID was mainly based on the Human Metabolome Database (www.hmdb.ca) and LIPID MAPS (www.lipidmaps.org); FC value was calculated as the ratio of the average mass response (area) between the two groups (FC value = PD/HC). P values less than 0.05 indicated significantly differences between the two groups. RT, retention time; VIP, variable influence on projection; FC, fold change; MS, matching score; FS, fragmentation score; ME, mass error; PD, Parkinson’s disease; HC, healthy control; P values have already been adjusted using the Benjamini and Hochberg method. Compound IDs underlined were shared differentially expressed metabolites in both sexes.

In the meantime, ten components were found in the PCA of the female comparison, with a R^2^X value of 0.693 and Q^2^ value of 0.416 (Fig. 1B). Further, the result of OPLS-DA also showed clear separations between healthy controls and PD patients (R^2^X = 0.173, R^2^Y = 0.729, Q^2^ = 0.406) (Fig. 1D, Supplementary Fig. 2B). The T-predicted scatter plot based on the OPLS-DA model generating with female metabolites demonstrated that male PD patients could not be effectively separated from male healthy controls, showing 37.5% of healthy controls were wrongly predicted as PD patients (Fig. 2B). There were 86 differentially expressed metabolites between the two groups (Table 3). Fatty acids and conjugates (23, 26.7%) and glycerophosphocholines (11, 12.8%) were significantly enriched (Fig. 3C, D). The differentially expressed glycerophosphocholines were LysoPC(22:0/0:0), PC(14:1/P-18:1), PC(P-20:0/22:4), PC(10:0/10:0), PC(O-1:0/16:0), PC(32:0), PC(O-16:0/18:0), PC(O-16:0/3:0), PC(36:0), PC(15:0/20:4), and PC(13:0), all of which had decreased except for PC(P-20:0/22:4).

There were 17 metabolites simultaneously changed in the male and female participants, with 10 increased metabolites (namely PS(12:0/15:0), p-Hydroxyphenylacetic acid, 2-tridecene-4,7-diynal, N-linoleoyl taurine, p-Cresol sulfate, p-Cresol, L-3-Phenyllactic acid, 13 S-hydroxyoctadecadienoic acid, Tephcalostan, and Chenodeoxycholic acid) and 6 decreased metabolites (including PA(P-16:0/18:4), LysoPC(22:0/0:0), PGF2alpha methyl ether, Aleb(d18:1/18:0), 2-methyl-dodecanedioic acid, and PG(19:0/22:2)). The level of eicosapentaenoic acid had increased in the female PD patients while decreased in the male patients.

## Discussion

Lipid metabolism is significantly correlated with PD. A meta-analysis including 15 cohort studies with 9740 participants confirmed that lower blood levels of triglyceride, total cholesterol, high-density lipoprotein cholesterol, and low-density lipoprotein cholesterol were associated with the occurrence of PD^13^. The interaction of α-synuclein with lipid membranes has been implicated in the formation of misfolding of α-synuclein into β-sheet-rich amyloid structures and subsequent aggregation, which is the probable pathogenesis of PD^14^. Sex differences were also found in very-low-density lipoprotein, triglyceride, and low-density lipoprotein cholesterol with age dependence^6^. Here, we were the first to perform liquid chromatography-mass spectrometry-based metabolomics profiling in PD patients to clarify sex differences and found some specific lipid changes as follows.

There was a total of 17 metabolites simultaneously changed in the male and female participants, and most of the metabolites showed consistent changes except for eicosapentaenoic acid. These metabolites can mainly be categorized into fatty acyls (namely PS(12:0/15:0), 2-tridecene-4,7-diynal, N-linoleoyl taurine, 13 S-hydroxyoctadecadienoic acid, Chenodeoxycholic acid, PA(P-16:0/18:4), LysoPC(22:0/0:0), PGF2alpha methyl ether, Aleb(d18:1/18:0), 2-methyl-dodecanedioic acid, and PG(19:0/22:2)) and benzenoids (namely p-Hydroxyphenylacetic acid, p-Cresol sulfate, p-Cresol, L-3-Phenyllactic acid, and Tephcalostan). Some plasma levels of fatty acyls had decreased and the others increased while all benzenoids had increased in PD patients. Based on the above 17 metabolites, no metabolite sets were enriched according to enrichment analysis and the detailed effects of these metabolites in PD need further researches to validate. The plasma levels of eicosapentaenoic acid in the male PD patients had decreased while increased in the female patients. Eicosapentaenoic acid is an important polyunsaturated fatty acid and can serve as the precursor for eicosanoids including the prostaglandin-3 and thromboxane-3 families. Eicosapentaenoic acid is probably a contributor to sex differences of eicosanoids in PD patients. According to enrichment analysis of all the differentially expressed metabolites in the male and female comparisons, the category of fatty acids and conjugates is the only enriched metabolite set in both male and female participants. Eicosanoids are the specific altered metabolite sets in the male while glycerophosphocholines are mainly enriched in the female participants.

### Fatty acids and conjugates

The category of fatty acids and conjugates is a subclass of fatty acyls, and elongation enzymes of very long-chain fatty acids and fatty acid metabolism played important roles in sexual differentiation of tambaqui^15^. The former gas chromatography-mass spectrometry analysis with urine samples of healthy participants indicated that saturated fatty acids were significantly different between sexes^16^. The males had significantly higher triglyceride levels and lower levels of monounsaturated fatty acids and eicosadienoic acids^17^. In this research, the category of fatty acids and conjugates was the only enriched metabolite set in both female and male participants between PD patients and healthy controls. Fatty acids and conjugates were the most primary differentially expressed metabolites, accounting for 20% in the male and 26.7% in the female. PD is reportedly associated with impaired gut-blood barrier for short-chain fatty acids^18^. α-Synuclein is the pathological hallmark of PD and can interact with lipids and fatty acids. In the meantime, only three metabolites belonging to fatty acids and conjugates were simultaneously changed between different sexes, of which N-linoleoyl taurine and 2-methyl-dodecanedioic acid showed consistent changes while the change of eicosapentaenoic acid was opposite. Most of the identified fatty acids and conjugates (7/11) in the male patients had decreased while the majority (18/23) in the female patients had increased. Therefore, significant sex differences were also found in fatty acids and conjugates among PD patients. Unsaturated fatty acids can be generated by hormone-sensitive lipase and the females had lifelong reduced lipase expression. Reducing the lipase in mutant α-synuclein mice improves Parkinson-like deficits through fatty acid metabolism especially in male mice^19^. Co-regulating fatty acid synthesis and degradation is a promising therapeutic strategy for PD patients based on different sexes^20^.

### Eicosanoids

The category of eicosanoids is also a subclass of fatty acyls derived from arachidonic acid, including prostaglandins, leukotrienes, hydroxyeicosatetraenoic acids, epoxyeicosatrienoic acids, and lipoxins^21^. Post-mortem analysis of the substantia nigra from PD patients demonstrated activation of microglia and increased levels of eicosanoids, leading to neuronal damage and clinical symptoms^22^. Eicosanoids and related enzymes (cyclooxygenases and cytochrome P450 enzymes) are new therapeutic opportunities for PD^5,23^. In this research, we found eicosanoids mainly participated in the male PD patients but not the female. PGF2alpha methyl ether is the only differentially expressed eicosanoids in the female and had simultaneously decreased in both sexes. The levels of Prostaglandin D3 and 11-dehydro-2,3-dinor-TXB2 had decreased in the male PD patients while levels of 9-deoxy-9-methylene-16,16-dimethyl-PGE2 and 20-carboxy-LTB4 had increased, indicating different effects on the development of PD. In previous research, sex differences were found in eicosanoid formation and metabolism of cardiovascular diseases and any other health conditions^24,25^. The eicosanoid production varies by sex^26^ and eicosanoid pathway is expressed throughout the testes. Prostaglandin is involved in processes of germ cell development and steroidogenesis in the male^27^.

### Glycerophosphocholines

Glycerophosphocholines (GPCs) are reportedly involved in depression, anxiety, dementia, and many other neurological symptoms. The decreased LysoPC(22:0/0:0) is the only differentially expressed GPCs in both sexes and the majority of GPCs have decreased in PD patients. Lysophosphatidylcholines (LysoPCs), hydrolysates from GPCs by phospholipase A2, can specifically bind to the G protein-coupled receptor family and induce intracellular calcium mobilization leading to increased glucose-stimulated insulin secretion. LysoPCs have several protective or anti-inflammatory effects and can serve as dual-activity ligand molecules in the innate immune system^28^. GPCs can serve as contributors of choline and phospholipid in central nervous system after crossing the blood-brain barrier. The various decreased GPCs are obviously correlated with the occurrence and development of PD. Although GPCs shared the similar percentages of differentially expressed metabolites (10.9% in the male and 12.8% in the female), they were only significantly enriched in the female participants as indicated by the enrichment analyses of metabolite sets. Sex-specific metabolic shifts were also found and GPCs were significant altered in the female non-severe COVID-19 patients^29^. We were the first to report sex-specific GPCs correlated with PD patients. GPCs might be ideal nutrients for female PD patients and it is essential for clinical researches to clarify it.

### Exogenous metabolites

Upon reviewing all the differentially expressed metabolites, some exogenous metabolites originate from animals or plants (including N-linoleoyl taurine, Alchornoic acid, p-Hydroxyphenylacetic acid, Tephcalostan, 11Z-Eicosenoic acid, 9,10-DHOME, Amorphigenin O-vicianoside, Stoloniferone F, 11-acetoxy-3β,6α-dihydroxy-9,11-seco-5α-cholest-7-en-9-one, Vitexin 3’’’,4’’’-Di-O-acetyl 2’’-O-rhamnoside, and PC(16:0/5:0(CHO))), and some are synthetic constructs probably through food additives or food indirectly. Although most of exogenous metabolites can be metabolized in the liver and the gut before appearing in circulation, a lot of plant/animal derivatives can still be found in blood or other tissues^30^. Some metabolites are newly reported in animals or plants and still categorized into exogenous metabolites in public databases nowadays. It doesn’t mean they really are “exogenous” metabolites for human forever in future. As we know, PD is an interplay of genetic and environmental factors and all these exogenous metabolites from foods or food additives may contribute to the occurrence and development of PD. Food additives are serious concerns all over the world, especially in China, and lead to many modern diseases. In addition, some exogenous metabolites originate from enteric microorganism (Mycolipanolic acid (C27), p-Cresol sulfate, p-Cresol, Chenodeoxycholic acid, and glycine conjugate). Enteric microorganism is regarded as “gut brain” and reported to be related with many central nervous system diseases, especially PD. Metabolites from enteric microorganism can disturb metabolic pathway after crossing intestinal mucosal barrier into plasma and then mediate the development of various diseases.

### Summary and limitation

After all, we are the first to demonstrate sex differences using metabolomics analysis in PD patients. Consistent with former researches, lipid metabolic disturbance is found to participate in the development of PD in this research, especially fatty acids and conjugates, while the detailed lipid fingerprint varies in different sexes. The components of eicosanoids are significantly changed in the male PD patients. Glycerophosphocholines are enriched in the female participants and most of glycerophosphocholines have decreased in female PD patients. The components of shared differentially expressed metabolites are complicated. Significant sex differences of lipid metabolism are found in PD patients according to those findings.

There are some limitations to this research. Firstly, the sample size is relatively small to some extent. Further researches with large sample size should be performed to validate these findings and clarify the detailed pathogenesis of lipid disturbance in the development of PD based on different sexes. Secondly, there are heterogeneities among the participants, including probable lifestyles and medication situation of PD patients. The metabolism of dopamine analogs and agonists may be different between sexes and a large sample size of drug naïve patients can exclude these effects.

## Methods

### Participants

A group of PD patients were recruited in Department of Neurology, the First Affiliated Hospital of Chongqing Medical University. The inclusion criteria were: (i) The diagnostic criteria based on the European Federation of Neurological Societies and the International Parkinson Movement Disorder Society’s European Section; (ii) Patients only taking dopamine analogs or dopamine receptor agonists without any lipid-lowering drugs (Supplementary Table 1). The exclusion criteria were: (i) Secondary Parkinson’s disease or Parkinson-plus syndrome; (ii) Patients suffering from tumor, heart failure, chronic obstructive pulmonary disease, nephritis, infectious diseases, or any other severe chronic diseases at the enrollment; (iii) History of stroke, brain surgery, head trauma, brain tumor, or any other neurodegenerative diseases^31^.

Healthy controls were included from Department of Physical Examination and they were without any history of illness in central nervous system or suffering from severe diseases. This study was approved by the Ethics Committee of the First Affiliated Hospital of Chongqing Medical University (2015-16) and performed in accordance with Declaration of Helsinki. Statements of informed consent were obtained from all the participants prior to inclusion in this study. Clinical characteristics and metabolomics analysis were blindly collected or performed^32^.

### Clinical characteristics

Clinical characteristics, including age, smoking history, alcohol consumption, hypertension, diabetes mellitus, hypercholesterolemia, and coronary heart disease, of all the participants were collected. All of PD patients were free of drug for more than 12 h and fast plasma samples were obtained by puncture of the median cubital vein at 6:00 am. The plasma samples were stored at -80℃ until analysis. The levels of total cholesterol, triglyceride, high-density lipoprotein cholesterol, low-density lipoprotein cholesterol, apolipoprotein A1, apolipoprotein B, and lipoprotein a were also measured using a Cobas Integra 400 plus automatic biochemical analyzer with matched reagent kits (Roche, Basel, Switzerland)^32^.

### Metabolomics analysis

We adopted a Waters Ultra Performance Liquid Chromatography I-class system equipped with a binary solvent delivery manager and a sample manager, coupled with a Waters VION IMS Q-TOF Mass Spectrometer equipped with an electrospray interface (Waters Corporation, Milford, USA) to perform the untargeted liquid chromatography-mass spectrometry-based metabolomics profiling. The detailed procedure of metabolomics analysis was described in former researches^2,32^. Generally, plasma samples stored at -80℃ were gradually thawed on ice, and 2-chloro-1-phenylalanine dissolved in methanol (0.3 mg/mL) was served as internal standard. Quality control sample was obtained by mixing all the samples in equal volume as a pooled sample and injected at regular intervals (every 10 samples) throughout the analytical run to provide a set of data from which repeatability can be assessed. Acquity BEH C18 column (100 mm×2.1 mm i.d., 1.7 μm; Waters Corporation) was used and the following gradients were used for separation: 5% B-25% B over 0–1.5 min, 25% B-100% B over 1.5–10 min, 100% B-100% B over 10–13 min, 100% B-5% B over 13–13.5 min, and 13.5–14.5 min holding at 5% B at a flow rate of 0.4 mL/min, where B is acetonitrile (0.1% (v/v) formic acid) and A is aqueous formic acid (0.1% (v/v) formic acid). The source temperature and desolvation temperature were set at 120℃ and 500℃, respectively, with a desolvation gas flow of 900 L/h. Centroid data was collected from 50 to 1,000 m/z with a scan time of 0.1 s and interscan delay of 0.02 s over a 13-minute analysis time. The obtained data were processed by baseline filtering, peak identification, integration, retention time correction, peak alignment and normalization using the build-in metabolomics software Progenesis QI (Waters Corporation). After that, data sets including m/z, peak retention time, and peak intensity of each ion were obtained, and further reduced by removing any peaks with missing values in more than 60% of the total samples. Metabolite identification was also performed by the build-in software Progenesis QI. We identified metabolites based on accurate mass, isotope pattern and MS/MS spectra against public databases, including Metlin (https://metlin.scripps.edu), and Human Metabolome Database (HMDB, http://www.hmdb.ca). The public databases were local databases built in the Waters metabolomics analytic system and included more obtainable information of metabolites for identification than online databases. The fragmentation score was used to assess the quality of second fragment of MS/MS spectra and the total score was 100. Matching score was automatically calculated by software Progenesis QI to assess the accuracy of metabolite identification and mainly based on three parameters including deviation of the mass (20 scores), second fragment of MS/MS spectra (20 scores), and isotopic distribution (20 scores) with a total score of 60. Finally, the metabolite with a highest matching score was recognized as the identified metabolite. All identified metabolites were level 2 according to the Metabolomics Standards Initiative and some original MS/MS plots of identified metabolites were provided as supplementary Figs. 3–9. The peak intensity was deemed as expression level of a metabolite^32^.

The positive and negative peak data were merged using normalization by median and log transformation (base 10), and multivariate statistical analyses were further performed by the SIMCA-P 13.0 software package (Umetrics, Umea, Sweden). The unsupervised PCA models were used to overview the distributions of total data while the OPLS-DA models were constructed to show statistical differences and identify differentially expressed metabolites between each two groups. The models were validated by 7-hold cross validations and 200-time response permutation tests. The constructed OPLS-DA models were further used to validate their differential abilities of PD patients based on samples from the opposite sex using the T-predicted scatter plots. Metabolites with variable influence on projection values (obtained from the OPLS-DA model) of greater than 1.0, fold change values of greater than 1.5 or lower than 0.67, and *p* values (obtained from Student *t* test and then adjusted using the Benjamini Hochberg method) of less than 0.05 were recognized to be differentially expressed. Metabolite set enrichment analyses were performed based on the above metabolites using MetaboAnalyst 5.0 (metaboanalyst.ca)^31,32^.

### Statistical analysis

Statistical analyses were completed using a commercially available software package (IBM SPSS version 22.0, New York, USA). Continuous data were expressed as means (standard deviations) and compared using Student *t* tests. Categorical data were exhibited as absolute numbers and percentages (%), and analyzed using Pearson *χ*^*2*^-tests or Fisher exact tests. *P* values were further adjusted using the Benjamini and Hochberg method and the adjusted *p* values less than 0.05 were considered statistically significant^33^.

## Electronic supplementary material

Below is the link to the electronic supplementary material.

Supplementary Material 1

Supplementary Material 2

## Data Availability

Mei-Xue Dong had full access to all the data in the study and takes responsibility for the integrity of the data as well as the accuracy of the data analysis. Please contact Mei-Xue Dong for data request.
