# Supplementary material for: Plasma metabolomics profiles indicate sex differences of lipid metabolism in patients with Parkinson’s disease
Source: Sci Rep. 2024 Dec 28;14:31262. doi: 10.1038/s41598-024-82674-3 (PMC11682129; doi:10.1038/s41598-024-82674-3)

**Supplementary Figure legend**

**Supplementary figure 1.** The OPLS-DA plot of both sexes in the two groups. OPLS-DA, orthogonal partial least squares-discriminant analysis; HC, healthy control; PD, Parkinson’s disease.

**
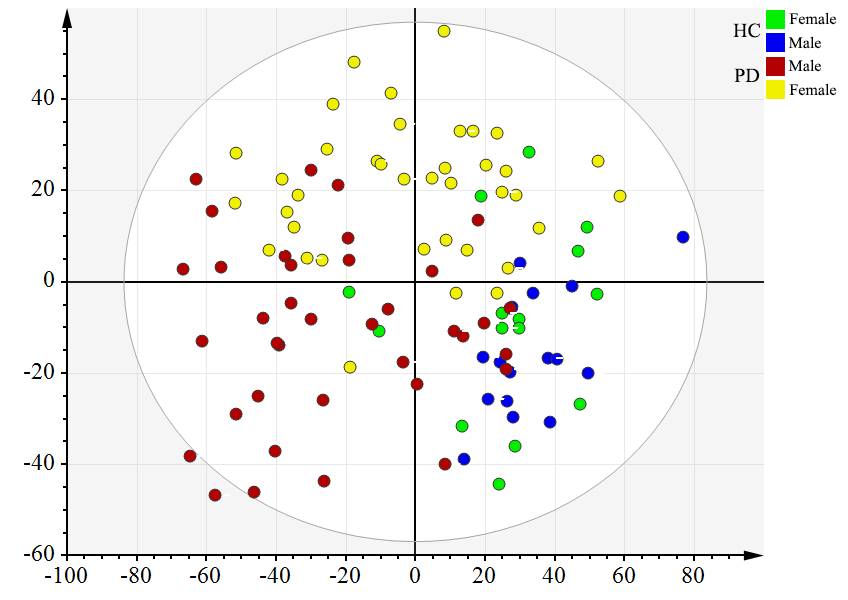
**

**Supplementary figure 2.** Statistical validations of the OPLS-DA models by response permutation testing of the male (A) and female (B) participants. The results indicated the two models were reliable without overfitting as the Q^2^ values were less than zero. OPLS-DA, orthogonal partial least squares-discriminant analysis.


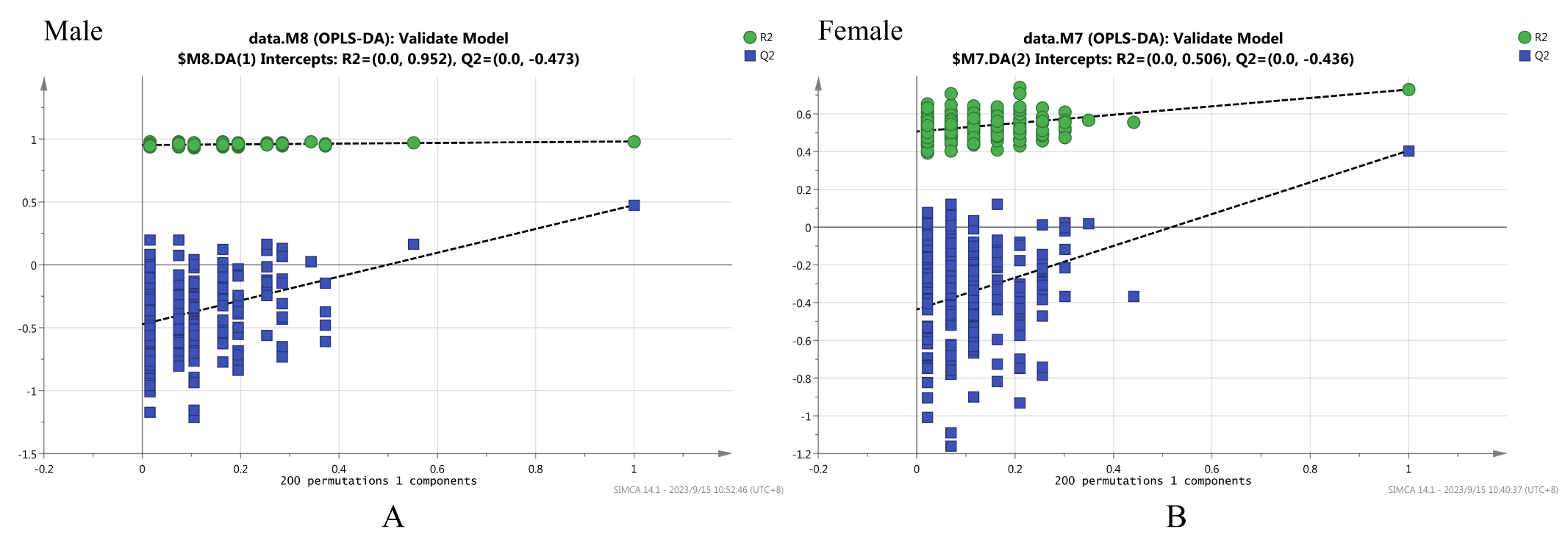


**Supplementary figure 3.** Original MS/MS plots of HMDB0001999.


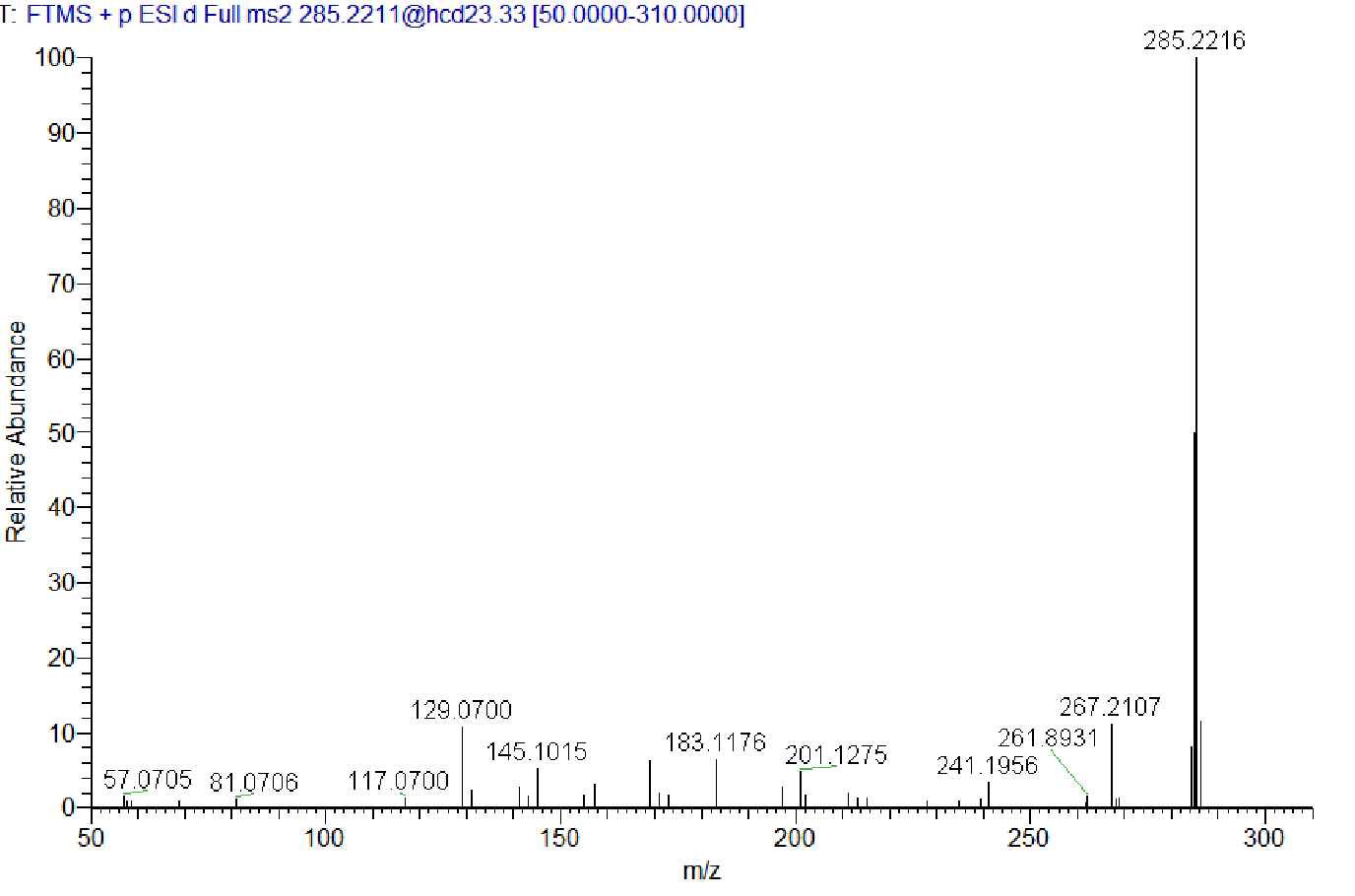


**Supplementary figure 4.** Original MS/MS plots of HMDB0010382.


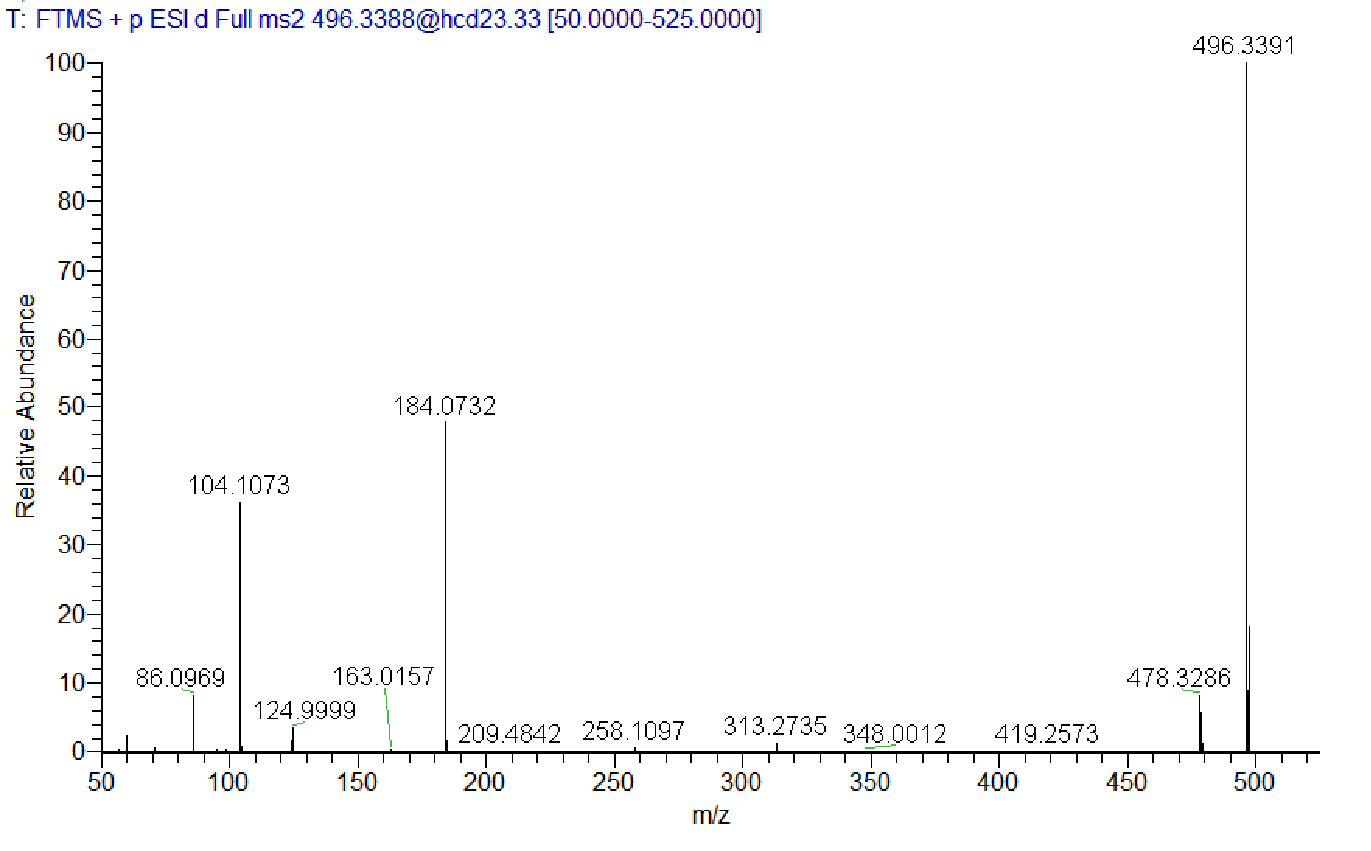


**Supplementary figure 5.** Original MS/MS plots of HMDB0011635.


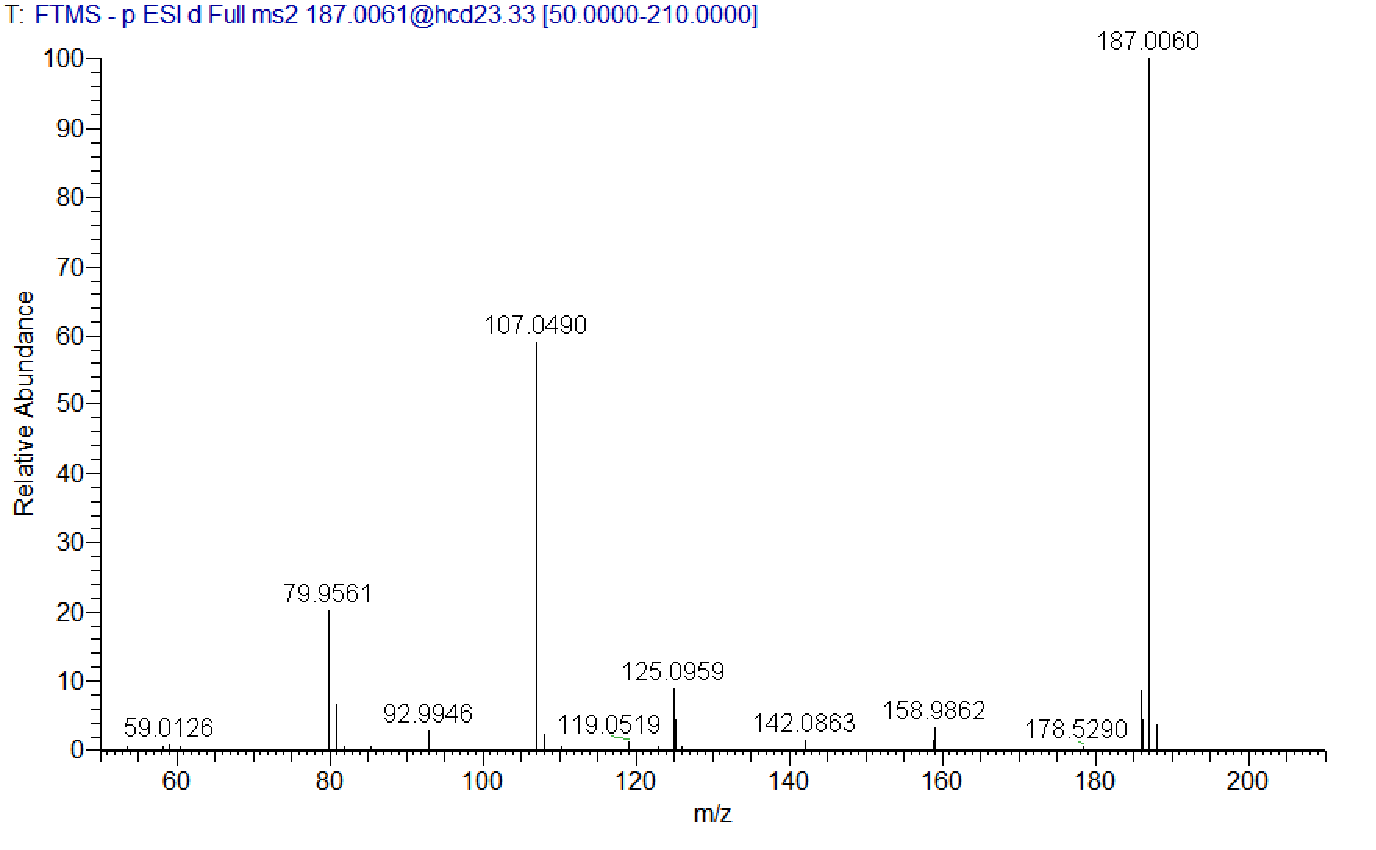


**Supplementary figure 6.** Original MS/MS plots of LMFA01170038.


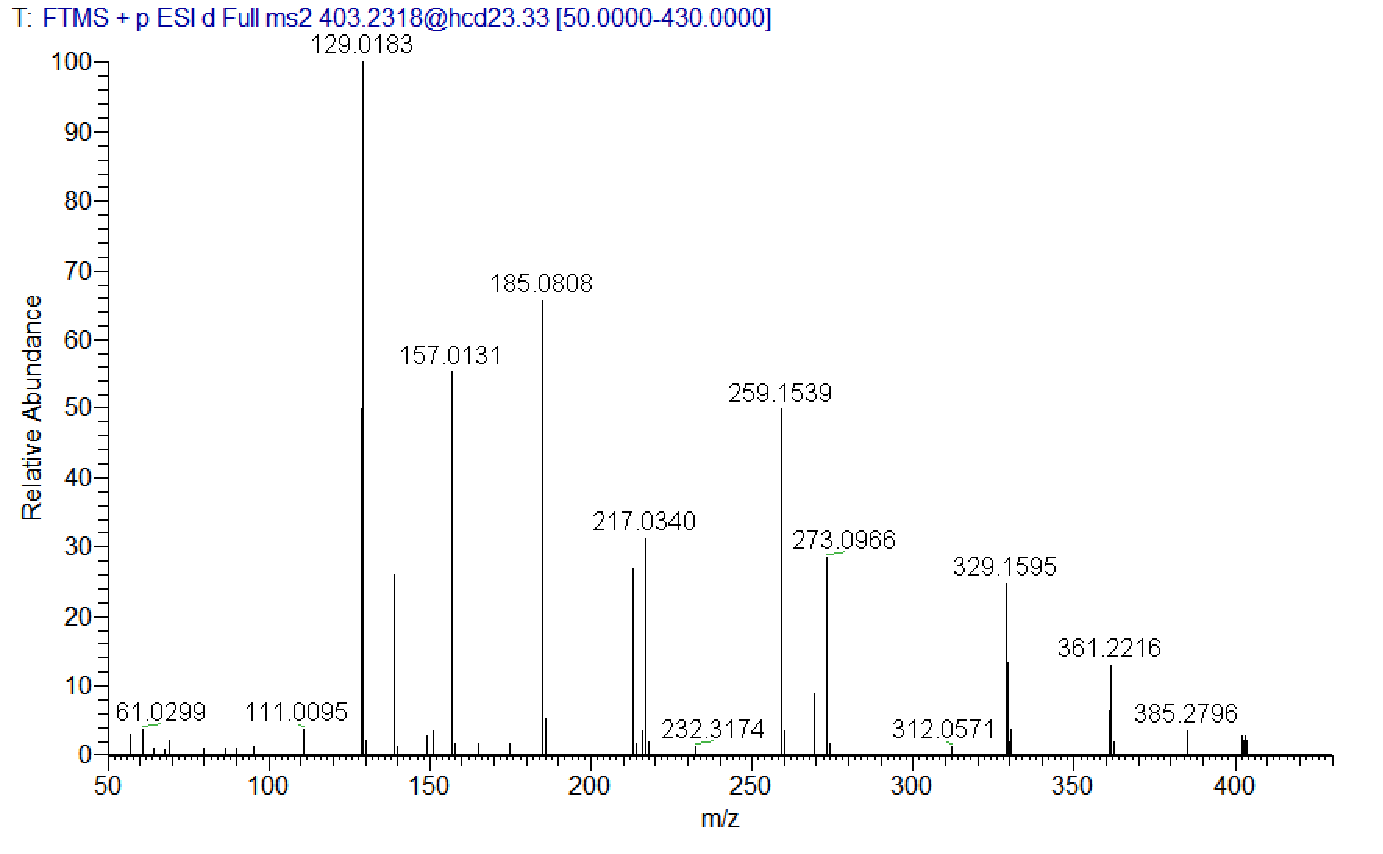


**Supplementary figure 7.** Original MS/MS plots of LMGP01020004.


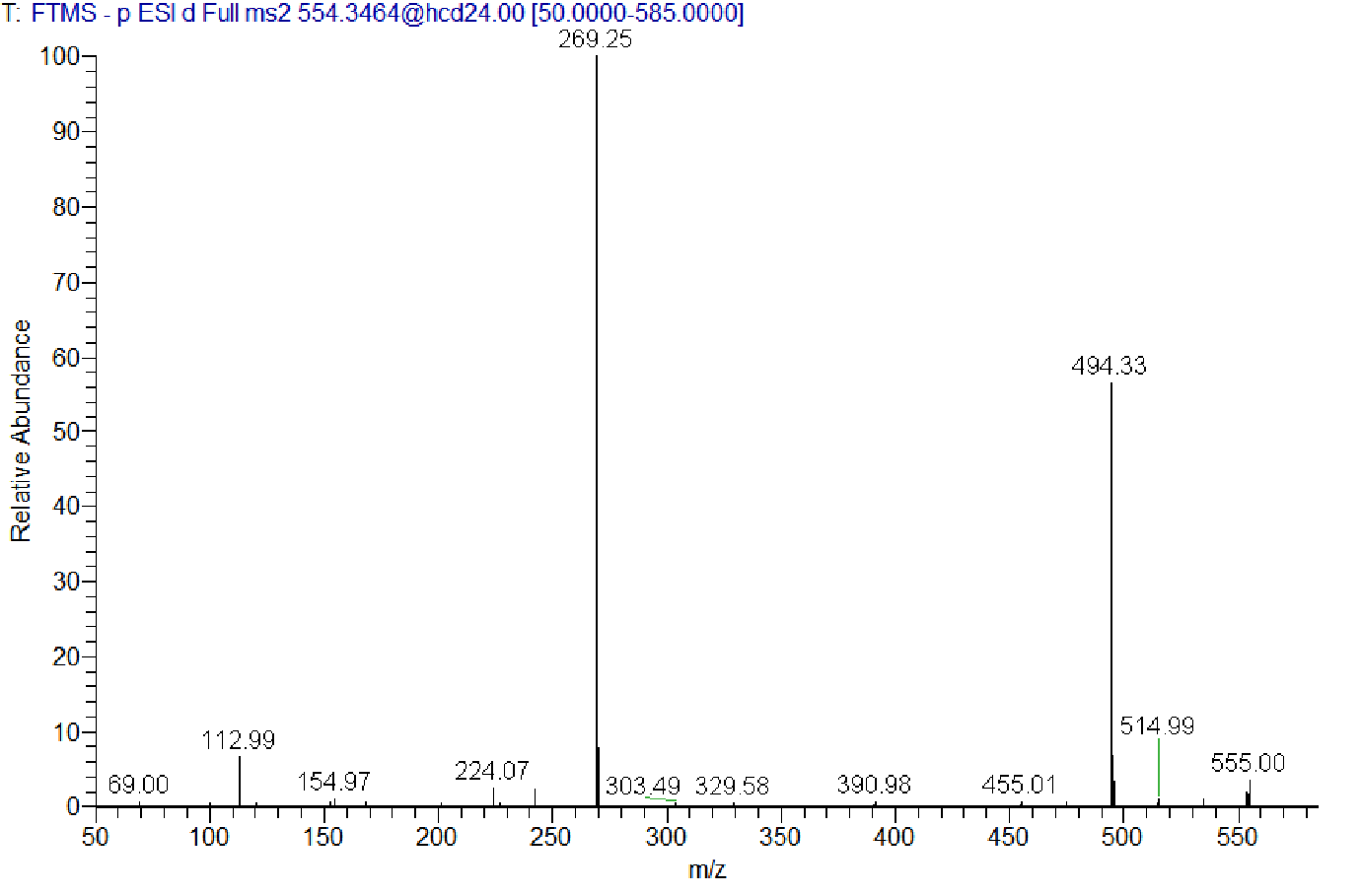


**Supplementary figure 8.** Original MS/MS plots of LMSP03010065.


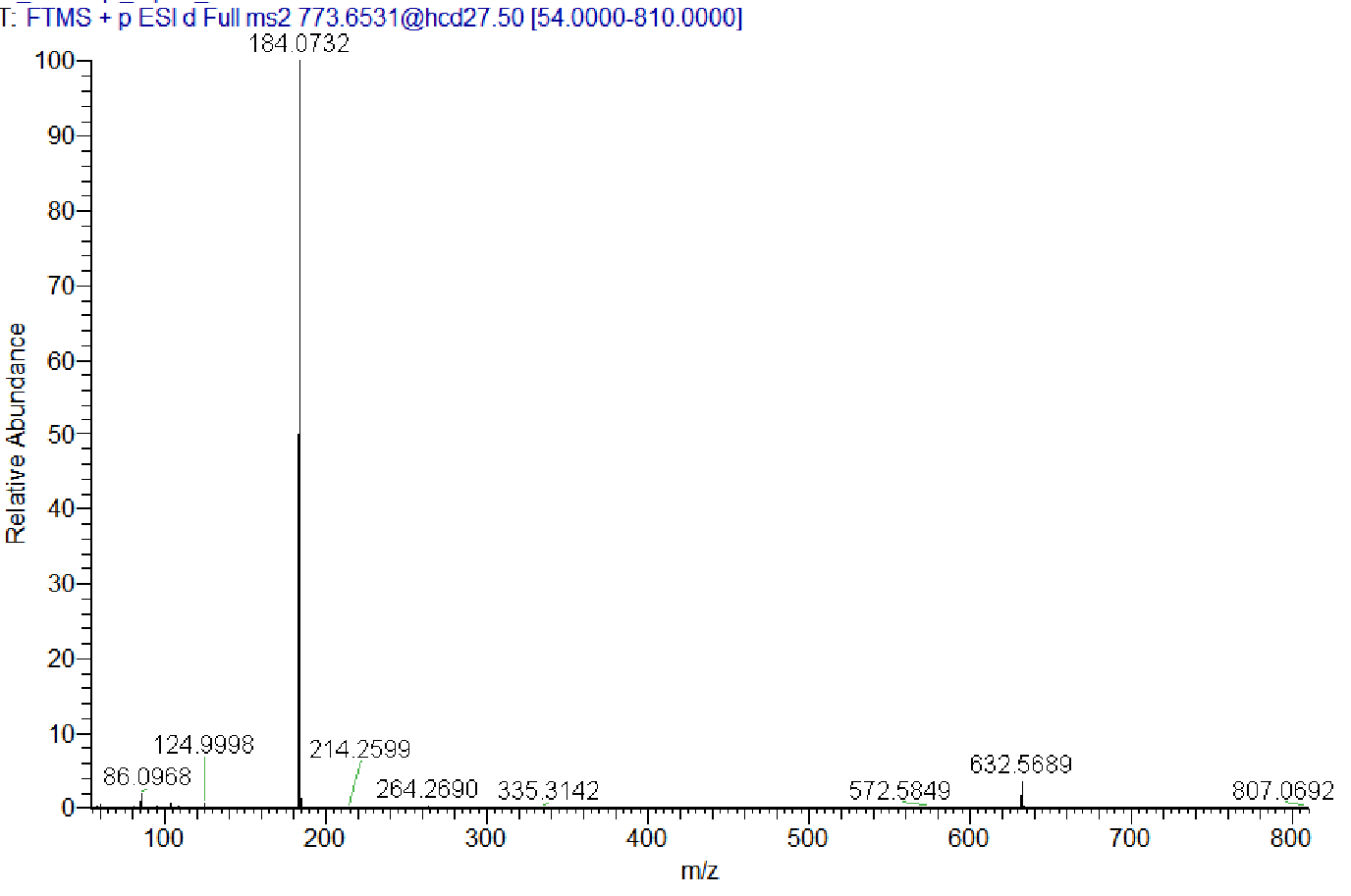


**Supplementary figure 9.** Original MS/MS plots of LMSP03010076.


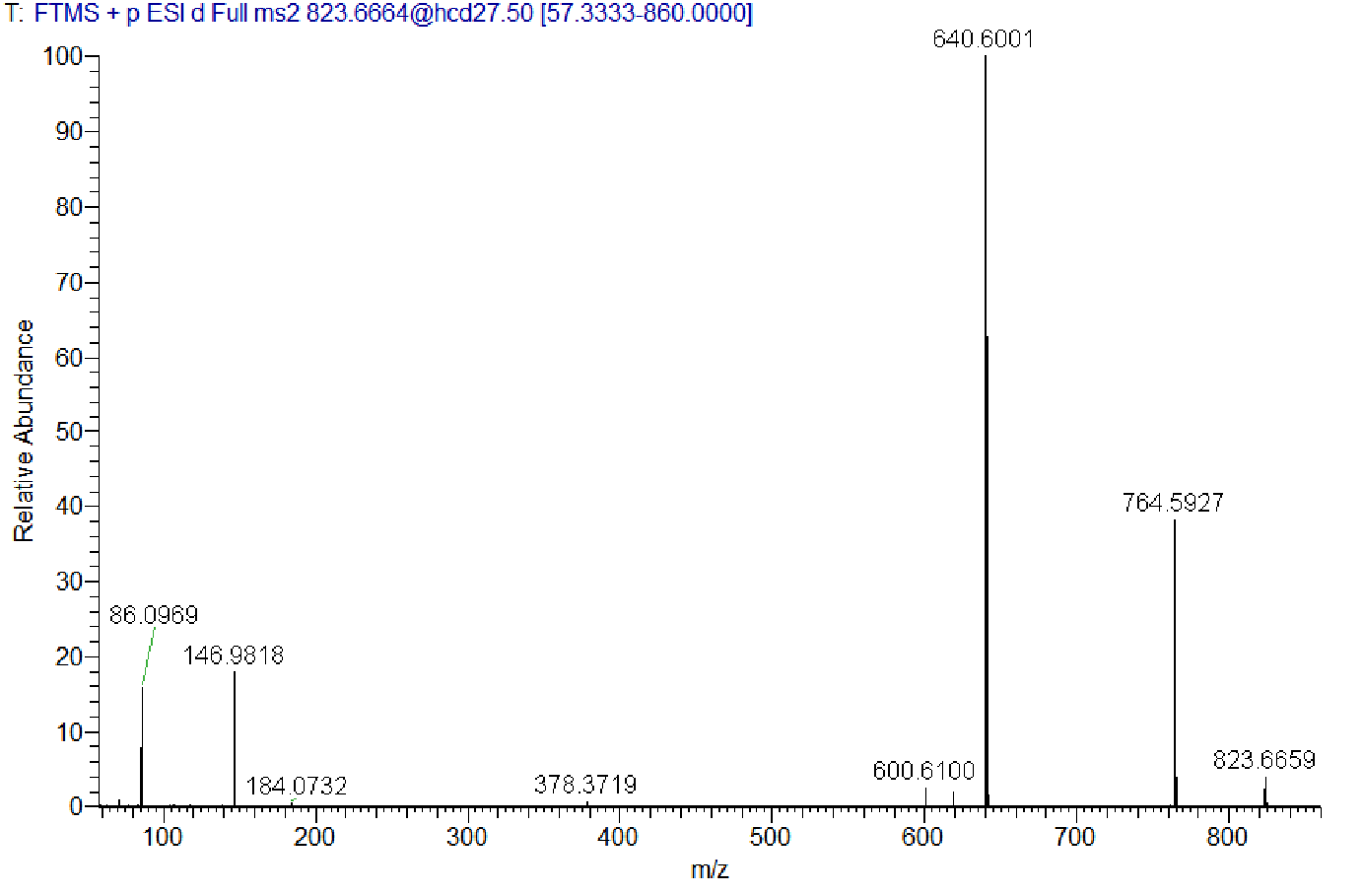

Supplement: Supplementary file 1 — Supplementary Material 1 [file 41598_2024_82674_MOESM1_ESM.docx]
