# Supplementary material for: Plasma metabolomics profiles indicate sex differences of lipid metabolism in patients with Parkinson’s disease
Source: Sci Rep. 2024 Dec 28;14:31262. doi: 10.1038/s41598-024-82674-3 (PMC11682129; doi:10.1038/s41598-024-82674-3)
Supplement: Supplementary file 2 — Supplementary Material 2 [file 41598_2024_82674_MOESM2_ESM.docx]

**Supplementary table 1.** The presentation of current drug taken of each PD patients included in this study.

| **Patient ID** | **Sex** | **Age** | **Disease duration （year）** | **Drug taken** | **Effect doses (mg)** |
| --- | --- | --- | --- | --- | --- |
| 1 | M | 69 | 4 | madopar 125mg tid | 300 |
| 2 | F | 78 | 12 | madopar 250mg tid, trastal SR 50mg bid | 700 |
| 3 | M | 70 | 11 | madopar 250mg tid, sifrol tablets 0.25mg tid | 675 |
| 4 | M | 58 | 7 | madopar 125mg tid, sinemet CR 125mg qid, sifrol tablets 0.5mg bid | 775 |
| 5 | M | 59 | 1 | None | 0 |
| 6 | M | 71 | 4 | madopar 250mg bid | 400 |
| 7 | F | 66 | 1 | madopar 250mg bid | 400 |
| 8 | F | 69 | 7 | madopar 125mg qid | 400 |
| 9 | M | 69 | 15 | madopar 250mg tid, trastal SR 50mg bid | 700 |
| 10 | M | 61 | 4 | None | 0 |
| 11 | M | 57 | 21 | madopar 125mg tid, sinemet CR 125mg qid | 675 |
| 12 | M | 73 | 15 | madopar 375mg tid, sifrol tablets 0.5mg bid | 1000 |
| 13 | F | 67 | 2 | madopar 125mg tid | 300 |
| 14 | F | 76 | 11 | None | 0 |
| 15 | F | 58 | 13 | madopar 125mg tid, trastal SR 125mg tid | 675 |
| 16 | M | 61 | 1 | madopar 125mg tid, sifrol tablets 0.25mg tid | 375 |
| 17 | M | 65 | 1 | trastal SR 25mg bid | 100 |
| 18 | M | 68 | 10 | madopar 250mg tid, trastal SR 50mg tid | 750 |
| 19 | M | 67 | 6 | None | 0 |
| 20 | M | 66 | 5 | madopar 187.5mg tid, trastal SR 50mg bid | 550 |
| 21 | F | 74 | 16 | sinemet CR 125mg qid | 375 |
| 22 | F | 66 | 3 | madopar 187.5mg tid, trastal SR 50mg bid | 550 |
| 23 | M | 72 | 14 | sinemet CR 312.5mg qid | 937.5 |
| 24 | M | 69 | 9 | madopar 250mg tid | 600 |
| 25 | F | 77 | 12 | madopar 250mg bid, sinemet CR 125mg qn | 493.75 |
| 26 | M | 72 | 2 | madopar 250mg bid, trastal SR 50mg qn | 450 |
| 27 | F | 66 | 4 | trastal SR 50mg bid | 100 |
| 28 | F | 50 | 3 | sinemet CR 125mg qid, trastal SR 50mg qid | 575 |
| 29 | F | 73 | 3 | madopar 250mg qd | 200 |
| 30 | F | 59 | 0 | None | 0 |
| 31 | F | 75 | 7 | madopar 125mg tid, trastal SR 50mg qn | 350 |
| 32 | M | 63 | 0 | None | 0 |
| 33 | M | 75 | 3 | madopar 250mg bid | 400 |
| 34 | M | 72 | 4 | madopar 250mg qid, trastal SR 50mg tid | 950 |
| 35 | M | 75 | 8 | madopar 250mg qid, trastal SR 50mg qid | 1000 |
| 36 | F | 62 | 9 | madopar 125mg tid, sifrol tablets 0.5mg tid | 450 |
| 37 | F | 65 | 5 | madopar 125mg tid, trastal SR 50mg tid | 450 |
| 38 | M | 74 | 1 | madopar 125mg bid | 200 |
| 39 | F | 71 | 4 | madopar 125mg qid, sifrol tablets 1.5mg qid | 1000 |
| 40 | M | 75 | 3 | madopar 125mg tid, trastal SR 50mg tid | 450 |
| 41 | M | 63 | 2 | None | 0 |
| 42 | F | 65 | 1 | madopar 125mg tid | 300 |
| 43 | F | 74 | 7 | madopar 250mg qid | 800 |
| 44 | F | 60 | 0 | None | 0 |
| 45 | F | 65 | 4 | madopar 125mg tid, trastal SR 50mg tid | 450 |
| 46 | M | 74 | 8 | madopar 250mg qid | 800 |
| 47 | F | 59 | 7 | madopar 250mg qid | 800 |
| 48 | F | 61 | 1 | None | 0 |
| 49 | F | 67 | 2 | madopar 125mg tid, trastal SR 50mg tid, sifrol tablets 0.5mg tid | 600 |
| 50 | M | 71 | 16 | madopar 125mg tid, trastal SR 50mg tid | 450 |
| 51 | M | 67 | 5 | madopar 125mg tid, sifrol tablets 0.5mg bid | 400 |
| 52 | M | 62 | 5 | madopar 125mg tid | 300 |
| 53 | F | 58 | 13 | madopar 125mg qid, trastal SR 25mg tid | 475 |
| 54 | M | 69 | 9 | madopar 125mg qid, sifrol tablets 0.25mg tid | 475 |
| 55 | M | 63 | 2 | madopar 125mg tid, trastal SR 50mg tid | 450 |
| 56 | F | 62 | 2 | madopar 125mg tid, trastal SR 25mg bid | 350 |
| 57 | F | 57 | 14 | madopar 187.5mg tid | 450 |
| 58 | F | 73 | 4 | madopar 62.5mg tid, sinemet CR 250mg tid | 712.5 |
| 59 | M | 58 | 2 | None | 0 |
| 60 | F | 72 | 8 | madopar 187.5mg qid, trastal SR 50mg bid | 700 |
| 61 | M | 72 | 30 | None | 0 |
| 62 | F | 58 | 10 | madopar 125mg qid, sifrol tablets 0.25mg tid | 475 |
| 63 | M | 72 | 6 | madopar 250mg tid, sifrol tablets 0.5mg tid | 750 |
| 64 | F | 58 | 4 | None | 0 |
| 65 | M | 65 | 2 | madopar 125mg tid | 300 |
| 66 | F | 64 | 5 | madopar 250mg tid, sifrol tablets 0.5mg tid | 750 |
| 67 | F | 63 | 4 | madopar 125mg tid, trastal SR 25mg tid | 375 |
| 68 | F | 67 | 8 | madopar 125mg tid, trastal SR 25mg bid | 350 |
| 69 | F | 67 | 1 | None | 0 |
| 70 | M | 61 | 6 | madopar 125mg qid, trastal SR 25mg qid | 500 |
| 71 | F | 58 | 6 | None | 0 |
| 72 | M | 65 | 2 | None | 0 |
| 73 | M | 50 | 7 | madopar 125mg qid | 400 |
| 74 | F | 64 | 5 | madopar 125mg qid, sifrol tablets 0.5mg qid | 600 |
| 75 | F | 63 | 4 | madopar 125mg tid, trastal SR 25mg tid | 375 |

PD, Parkinson’s disease; F, female; M, male.
